# Supplementary material for: S-methyl Methanethiosulfonate: Promising Late Blight Inhibitor or Broad Range Toxin?
Source: Pathogens. 2020 Jun 22;9(6):496. doi: 10.3390/pathogens9060496 (PMC7350374; doi:10.3390/pathogens9060496)
Supplement: Supplementary file 1 [file pathogens-09-00496-s001.pdf]

Table S1. Growth parameters obtained for Bakers' yeast grown at different MMTS concentrations.

MMTS Treatment	Carrying Capacity k [OD 600]	Growth Rate Constant r	Time at Inflection Point [h]
DMSO Control	0.927	0.459	7.855
1.95 µg/mL	0.919	0.528	7.685
3.9 µg/mL	0.901	0.591	9.069
7.8 µg/mL	0.881	0.465	11.988
15.6 µg/mL	0.005	0.241	3.463

Table S2. Minimal inhibiting concentrations (MICs) of MMTS for wildtype bacterial species and clinical bacterial species carrying different antibiotic marker genes. MICs were determined in cation-adjusted Muller-Hinton broth (CA-MHB). A mother-solution of MMTS of 256 µg/mL was prepared by dissolving MMTS in water and serial dilutions were obtained by mixing with CA-MHB in 96 well plates. A range of concentrations of 0.125 µg/mL–128 µg/ml was tested against fresh bacterial colonies adjusted to final densities of roughly 10^6 CFU/mL in CA-MHB. The experiment was carried out in quadruplicates for each bacterial strain. Plates were sealed with parafilm and incubated at 37 °C for 16 h. Growth +/- was visually assessed after incubation.

Species	Resistance Phenotype and Determinant	MIC MMTS (µg/mL)
E. coli	WT	16
E. coli	Plasmid-mediated Colistin Resistance MCR-1	32
E. coli	Carbapenemase OXA-48, Extended spectrum ß-lactamase CTX-M-9	32
K. pneumoniae	Carbapenemase OXA-48	32
K. pneumoniae	Cephalosporinase DHA	32
A. baumannii	Colistin Resistant (chromosomally encoded)	8
A. baumannii	WT	16
P. aeruginosa	Colistin Resistant (chromosomally encoded)	16
C. freundii	Extended spectrum ß-lactamase CTX-M-1	32
S. aureus	Methicillin Susceptible	32
S. aureus	Methicillin Resistant MecA (MRSA)	16
S. aureus	Daptomycine Resistant	32
E. faecalis	Vancomycin Susceptible	64
E. faecalis	Linezolide Resistant, Vancomycin Resistant VanA	32 – 64
E. faecalis	Linezolide Resistant, Vancomycin Resistant VanA	32
E. faecium	Linezolide Resistant, Vancomycin Resistant VanA	64
E. faecium	Linezolide Resistant	32 – 64
S. epidermidis	Linezolide Resistant	16

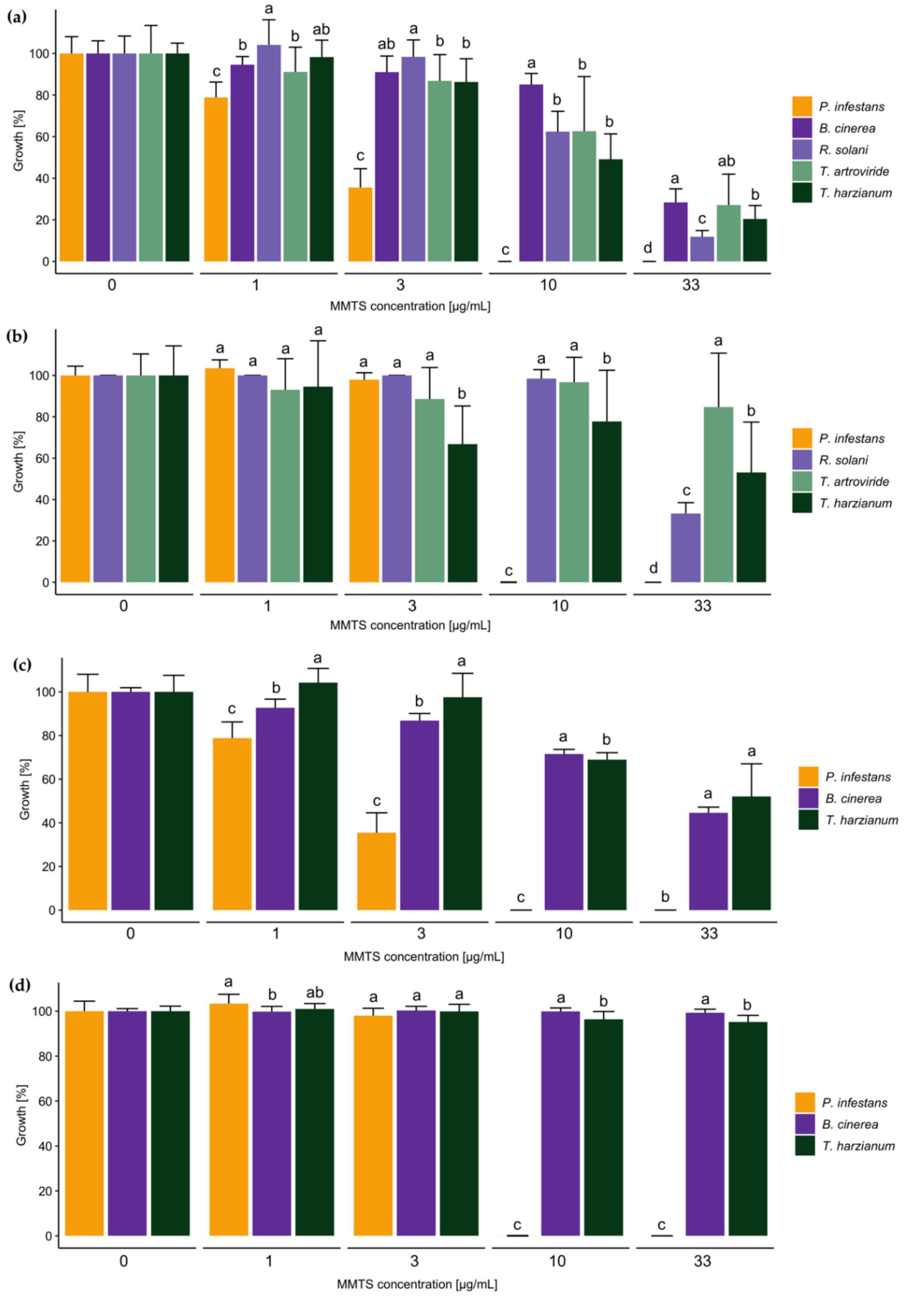

Figure S1. Significant differences in growth area inhibition of mycelial growth of *P. infestans* on V8 medium and, *T. harzianum*, *T. artroviride*, *R. solani*,*B. cinerea* grown on PDA medium (**a**), (**b**) as well as *R. solani* and *B. cinerea grown* on V8 instead of PDA compared to *P. infestans* (**c**), (**d**) at MMTS concentrations of 1–33 µg/mL. Growth area quantifications in percentage to the solvent control are shown at an early timepoint; (**a**), (**c**) and a later timepoint; (**b**), (**d**). Average growth compared to the solvent control (100%) depicted in the upper graphs corresponds to the mean of 8 replicates pooled from two independent experiments with four replicates each. Error bars represent standard deviation (n = 8). Letters indicate significant differences in growth area percentages between fungi and

oomycete at a given MMTS concentration according to ANOVA and LSD (Least Significant Difference) for post hoc analysis ($p < 0.05$).

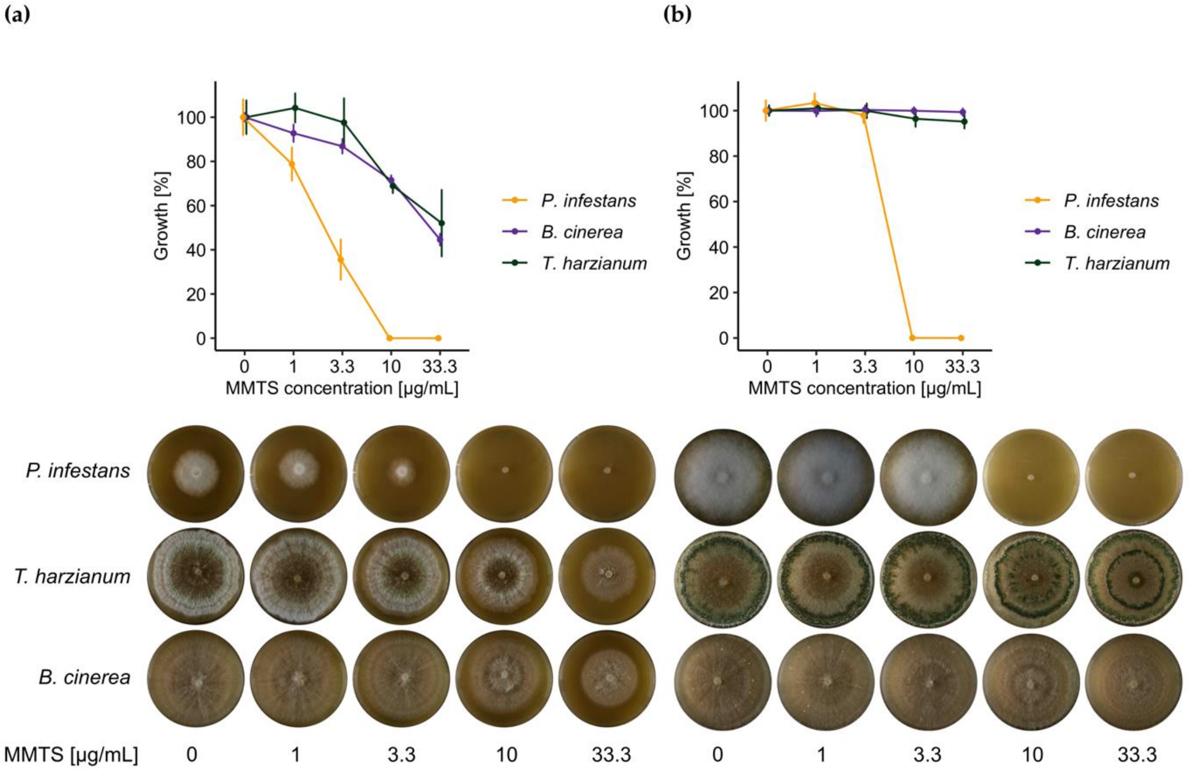

Figure S2. Inhibition of mycelial growth of *P. infestans*, *T. harzianum* and *B. cinerea* grown on V8 medium with added MMTS at concentrations of 1–33 µg/mL. Representative pictures and corresponding growth area quantifications in percentage to the solvent control are shown at an early timepoint: (**a**) and a later timepoint; (**b**). Timepoint 1 corresponded to 4–7 days after inoculation, Timepoint 2 to 12–14 days after inoculation (depending on mycelial growth speed). Average growth compared to the solvent control (100%) depicted in the upper graphs corresponds to the mean of 8 replicates pooled from two independent experiments with four replicates each. Error bars represent standard deviation (n = 8). Significant differences in growth percentages at a given MMTS concentration between fungal species and *P. infestans* are shown in Figure S1cd.

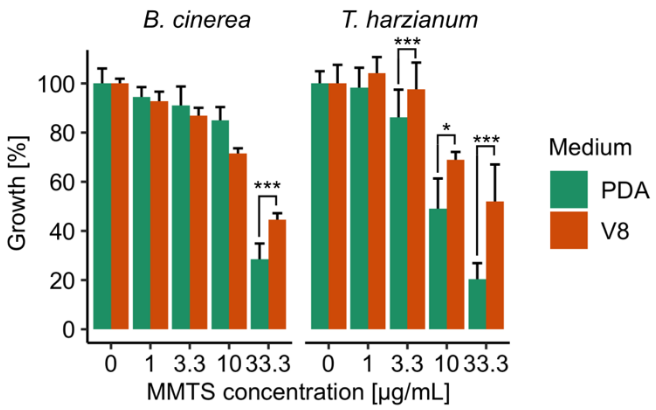

Figure S3. Significant differences in inhibition of mycelial growth of *T. harzianum* and *B. cinerea* grown on V8 or PDA medium with added MMTS at concentrations of 1–33 µg/mL. Growth area quantifications in percentage to the average control are shown at an early timepoint. Bars in graphs

correspond to average growth percentage of 2 repetitions with 4 replicates each. Error bars represent standard deviation. Asteriks indicate significant differences in growth area percentages between media for a given MMTS concentration and fungus according to ANOVA and Tukey's HSD for post-hoc analysis (significance levels: *p*-values: ≤0.001 ***, ≤0.01 **, ≤0.5 *).

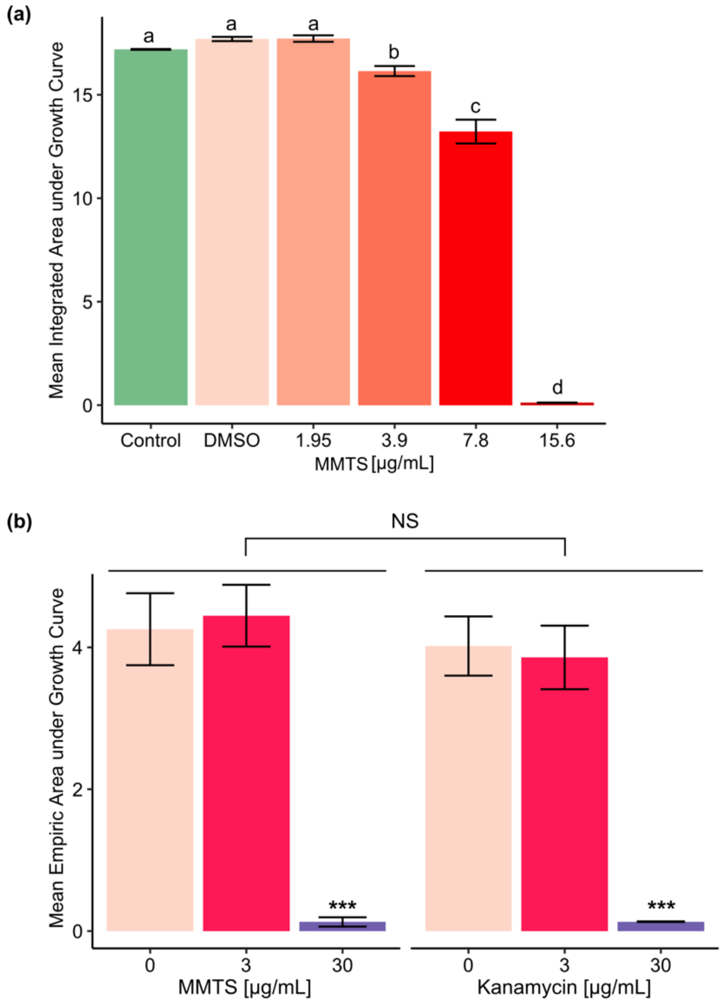

Figure S4. Significant differences between growth curves of Bakers' yeast (**a**) treated with 1.95–3.9 µg/mL MMTS and *D. solani* (**b**) treated with 0–30 µg/mL MMTS or kanamycin. (**a**): The average integrated area under the growth curve, calculated by fitting a log-logistic model to the data, corresponds to the mean of 3 replicates (*n* = 3). Letters indicate significant differences between integrated areas according to ANOVA followed by LSD for post-hoc analysis. (**b**); The average empiric area under the growth curve, calculated by summing up the trapezoids connecting the Data points under growth curves, corresponds to the mean of 3 replicates (*n* = 3). A two-way ANOVA followed by Tukey's HSD for post-hoc analysis was applied to the data to calculate significant differences between growth curves of *D. solani* exposed to different concentrations of MMTS and kanamycin (significance levels: *p*-values: ≤0.001 ***, ≤0.01 **, ≤0.05 *).

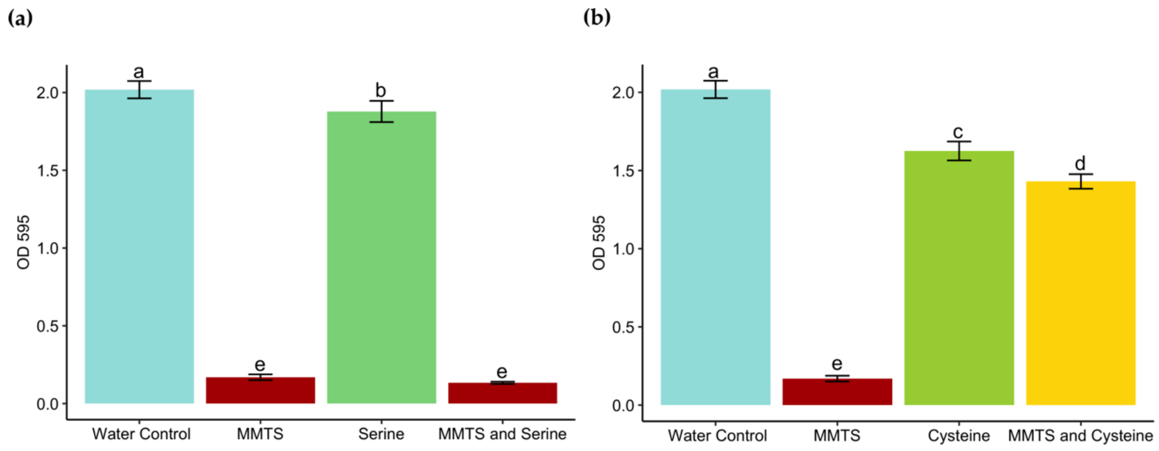

Figure S5. Trapping of MMTS by cysteine in the growth medium. A fresh starter culture of *D. solani* in LB was diluted with LB in test tubes to an OD_{595} of 0.05. MMTS, cysteine, serine, MMTS and cysteine or MMTS and Serine were then added to a final concentration of 8×10^{-7} mol/mL of each compound, which corresponds—for MMTS—to approximately 100 µg/mL. This concentration was chosen because 100 µg/mL of MMTS alone completely inhibits bacterial growth. Mother solutions of MMTS, cysteine and serine were realized in water. Bacterial cultures were incubated over night at 28 °C and 180 rpm. OD was measured after incubation. Panels (**a**) and (**b**) show the same data but split into two graphs for better visualization, (**a**) showing the effect on bacterial growth of serine added additionally to MMTS and (**b**) showing the effect of cysteine added additionally to MMTS. Bars represent the average OD of 2 repetitions with 3 replicates each with error bars corresponding to standard error (n = 6). Letters indicate significant differences between treatments according to ANOVA and Tukey's honest significant difference for post hoc analysis (p-value < 0.05).
